# Tenogenic Cues Are Biochemically and Environmentally Distinct for Tendon Stem Cells and Mesenchymal/Stromal Stem Cells

**DOI:** 10.1155/sci/9047956

**Published:** 2025-05-13

**Authors:** Vera Citro, Marta Clerici, Giovanna Della Porta, Nicola Maffulli, Aldo R. Boccaccini, Tina P. Dale, Nicholas R. Forsyth

**Affiliations:** ^1^School of Pharmacy and Bioengineering, Keele University, Stoke-on-Trent, Staffordshire ST4 7QB, UK; ^2^Institute of Biomaterials, Department of Materials Science and Engineering, University of Erlangen-Nuremberg, Erlangen 91058, Germany; ^3^Department of Medicine, Surgery and Dentistry, University of Salerno, Baronissi 84081, Italy; ^4^Interdepartmental Centre BIONAM, University of Salerno, Fisciano 84084, Italy; ^5^Department of Trauma and Orthopaedic Surgery, University Hospital “San Giovanni di Dio e Ruggi D'Aragona”, Salerno 84131, Italy; ^6^Department of Trauma and Orthopaedics, Faculty of Medicine and Psychology, Sant'Andrea Hospital, Sapienza University, Rome 00189, Italy; ^7^Vice Principals' Office, Kings College, University of Aberdeen, Aberdeen AB24 3FX, UK

## Abstract

Tendon tissue engineering draws on regenerative medicine principles, offering innovative solutions to address the challenges posed by tendon injuries and degenerative conditions. Tendons' inherent limited regenerative capacity often hinders complete recovery from injuries, leading to chronic conditions and impaired functionality. Autologous mesenchymal/stromal stem cells (MSCs) and tendon-derived stem cells (TSCs), combined with growth factors (GFs) like GDF-5, GDF-6 and GDF-7, are emerging as potential therapies for tendinopathy. These GFs are crucial for tendon development and promoting tenogenic differentiation, though the exact pathways they activate remain unclear. For this reason, directly comparing all three pathways to assess their impact on both MSCs and TSCs is essential. This study examined the effects of GDF-5, GDF-6 and GDF-7 on tenogenic differentiation in MSCs and TSCs, with a focus on how oxygen levels (21% O_2_ vs. physoxia at 2% O_2_) influence this process. The expression profiles of key tenogenic genes (Scleraxis [Scx], Tenomodulin [Tnmd], Thrombospondin-4 [Thromb-4] and Tenascin-C [Tnc-C]) were explored by quantitative reverse transcription PCR (RT-qPCR) following supplementation with individual GFs. Transcriptional analysis was complemented by Tnmd immunofluorescence (IF) and image analysis to identify optimal differentiation parameters. The study highlighted GDF-7 as a powerful inducer of tenocyte-like cell differentiation in MSCs, showcasing sustained expression of tenogenic genes over time in 21% O_2_. Moreover, TSCs in physoxia differentiate into tenocytes without an additional GF requirement. In conclusion, the study lays a foundation for understanding the complex interplay of GFs, oxygen levels and cellular responses in the quest for tendon regeneration. In doing so, it establishes that different cell types have differing biochemical requirements for induction of tenogenic differentiation. While offering promising avenues for tissue engineering platforms, it underscores the need for further research to fully harness the potential of MSCs and TSCs in vivo for tendon regeneration.

## 1. Introduction

In musculoskeletal consultations, injuries to tendons and ligaments make up 30% of cases, resulting in 17 million new occurrences globally each year [1]. This places a substantial burden on both the society and the economy with an estimated cost of over £30 billion per annum [2]. Injuries to tendons and ligaments, if not promptly and adequately addressed, can significantly impede normal body movement and give rise to various complications, such as pain, lack of mobility and even arthritis [1]. Traditional treatments involving surgical repair and tissue grafts can prove ineffective, often resulting in a high recurrence rate [3]. Regenerative medicine seeks to stimulate the natural regeneration of tissues in vivo or, via tissue engineering, to create functional tissue replacements in vitro for subsequent implantation into the body. As part of this approach, the manipulation of biochemical and physical factors has the potential to influence both cell behaviour and the production and organization of the extracellular matrix. The optimal combination of these factors, applied in a dosage- and time-dependent manner, is likely a crucial factor in the in vitro engineering of tendons.

Historically, tendons were thought to exclusively house tenocytes, responsible for the maintenance, repair and remodelling of tendons. There have been clinical trials reporting the use of tenocytes [4, 5] for the treatment of tendinopathy with promising results. Wang et al. [6] conducted a pilot study on the treatment of chronic resistant lateral epicondylitis, with autologous tenocyte injection, to counter the depletion of tenocyte populations in end-stage tendinopathy, caused by apoptotic and autophagic cell death. The concept behind this study was to provide a new pool of stable cells, which could synthesize new extracellular matrix resulting in the repair of tendon damage.

With the advancement of research, three main problems have been identified with the use of tenocytes: (1) tenocytes are highly differentiated cells and have limited capacity to replicate and differentiate, (2) phenotypic drift and function loss are often observed during in vitro expansion of tenocytes, and (3) tendons are relatively acellular and contain few tenocytes with the problem of donor site morbidity in autologous transplantation. To overcome these problems, the use of multipotent stem cells has been suggested. The application of stem cells helps regulate inflammation, organize ECM regeneration and promote tissue regeneration over scarring [7].

Mesenchymal/stromal stem cells (MSCs) have been widely studied and applied in regenerative medicine. Employing MSCs can deliver the dual benefits of a cell population with direct differentiation potential, as well as altered inflammatory setting, striving for a shift from cellular responses that are proinflammatory and profibrotic to those that are proregenerative. This anticipated change is expected to decrease the infiltration of inflammatory cells and encourage the orderly deposition of ECM components [8]. Three key properties of MSCs make them well-suited for tissue regeneration: their ability to regulate the immune system [9], which helps mitigate abnormal immune responses, their capacity for paracrine or autocrine functions that produce growth factors (GFs) [10] and their capability to differentiate into specific target cells [11]. Autologous and allogeneic MSCs have been explored for clinical applications in tissue regeneration [12]. Bone marrow MSCs were reported to stimulate biological and biomechanical healing of patellar tendon after injury [13]. Additionally, MSCs have been shown to support tendon repair by modulating macrophage phenotype, from proinflammatory to anti-inflammatory, and releasing bioactive molecules with immunosuppressive properties [14]. Despite these advantages, there are also cases in which use of MSCs induced formation of ectopic bone, after transplantation [15, 16]. Clarke et al. [17] pointed out the potential dangers of using undifferentiated cells that could not develop into the intended cell type, which could harm the native tissue. From this, it has been hypothesized that driving the MSCs toward the tenogenic lineage prior to transplantation may reduce the chance of ectopic bone and tumour formation as well as promote better tendon repair. In favour of this theory, MSC pretreatment with GFs induced the expression of key transcription factors, such as Scleraxis (Scx) and Mohawk (Mkx), to promote tenogenic differentiation of stem cells prior to delivery [18, 19].

Contemporary understanding also acknowledges the presence of various cell populations within tendons, including multipotent cells known as tendon-derived stem cells (TSCs). Despite challenges in characterizing these cells, including the lack of identification of tendon-specific markers, the environment or niche where TSCs are located plays a crucial role in providing signals that support the enduring stem cell properties of TSCs [20]. Like MSCs, TSCs have the potential for self-renewal and multidirectional differentiation [21]. Distinct from other stem cells, TSCs express higher levels of tendon-related genes (e.g. Scx, Tenomodulin [Tnmd], Tenascin-C [Tnc-C] and Thrombospondin-4 [Thromb-4]) [22]. Recently, investigators found that TSC therapy significantly accelerated tendon healing and had an effect in all three phases of tendon repair, in addition to increasing the ultimate strength of the repaired tendon [23]. A direct comparison showed that TSCs were a superior cell source compared to MSCs for musculoskeletal tissue regeneration. However, using TSCs for tissue repair requires an allogeneic cell source due to challenges in obtaining autologous TSCs without causing donor site issues [24].

Irrespective of the selected cell type, to achieve proper differentiation, it is essential to optimize the inductive signalling events guiding stem cells towards specific lineages. Unlike chondrogenic and osteogenic differentiation [25–27], there is no universally accepted induction protocol for tenogenesis. Typically, the addition of various GFs to the culture medium is employed to induce the desired phenotype, leveraging the potent regulatory effects of these biomolecules on biological responses [28]. Employing bioactive molecules in tenogenesis could hold significant importance for the initial stages of tendon tissue engineering, providing a bioactive environment that resembles the initial developmental phases of the tendon. It is crucial to assess various GFs to determine the most effective combination for promoting tenogenic differentiation.

Bone morphogenic proteins (BMPs) are members of the transforming GF beta superfamily, a group of GFs known to influence growth and differentiation of various cell types [29]. Originally, they were identified as components of de-mineralized bone that were potent inducers of bone and cartilage, both in vivo and in vitro [30]. Since the regeneration of adult tissue often mirrors developmental processes, insights from developmental biology can play a vital role in the context of tendon healing and restoration. Based on sequence similarity, BMPs have been grouped into several subtypes, and between them, we can select a specific subset, including BMP12 (GDF-7), BMP13 (GDF-6) and BMP14 (GDF-5) that are specifically recognized for their ability to induce tenogenic differentiation [19, 31–36].

Here, we examine the individual influence of GFs GDF-5, GDF-6 and GDF-7 on two successive developmental cell lines, namely, MSCs and TSCs. The objective is to discern the specific phases in which the influence of these GFs becomes predominant and to elucidate how, in synergy with oxygen concentration, they can intricately govern the tenogenic differentiation process.

## 2. Materials and Methods

### 2.1. Isolation and Culture of MSCs

Human bone marrow-derived mesenchymal stromal cells (BMSCs) have shown remarkable potential for tenogenic differentiation and tendon repair, positioning them as a key focus in regenerative medicine and advancing towards clinically relevant applications [13, 14, 37]. For this study, human bone marrow MSCs were isolated from commercially sourced donor bone marrow aspirate (Poietics Lonza, USA) retrieved from the iliac crest of three healthy donors aged between 18 and 45. Bone marrow aspirates were transported under controlled temperature conditions (−20°C) and supplemented with heparin to prevent coagulation. Seeding was performed within 48 h of aspiration where previous findings detail 80%–90% mononucleated cell (MNC) viability [38]. MSC isolation and expansion were performed using a modification of a previously described protocol [39]. Whole bone marrow was seeded at a density of 1 × 10^5^ MNC/cm^2^ on 10 ng/mL fibronectin-coated flasks in Dulbecco's Modified Eagles Media (DMEM, 4.5 g/L glucose, Corning) supplemented with 5% human platelet lysate (hPL, StemCell Technologies), 100 U/mL penicillin–streptomycin–amphotericin B, 1% NEAA and 1% L-glutamine (all Lonza, UK). After 7 days, half of the culture medium was replaced with fresh media; after 14 days, the media were replaced completely.

The recovery of MSCs was carried out in two different environments: either in humidified incubators with an oxygen concentration of 21% with 7% CO_2_ or in an Invivo2 workstation (Baker Ruskinn) maintained at 37°C with 7% CO_2_ and 2% O_2_ concentration. This commercially available system ensures appropriate oxygen concentration within the settled parameters, as demonstrated by previous works [40–43]. For reduced oxygen culture, the media were deoxygenated (HypoxyCOOL, Baker Ruskinn) to bypass inconsistencies associated with preincubation of media in hypoxic conditions. On days 7, 14 and 21, the culture flasks were washed with phosphate-buffered saline (PBS), fixed with 10% neutral buffered formalin and subjected to Giemsa staining (1:20 solution in distilled water). Clusters of cells stained positively with Giemsa, containing more than 16 cells, were considered as colony-forming unit-fibroblastic (CFU-F).

### 2.2. Isolation and Culture of Porcine TSCs

Swine are widely used in translational research, surgical models and preclinical pharmaceutical testing as an alternative to dogs or monkeys [44]. Their anatomical and physiological similarities to humans, particularly in intra-articular cartilage and ligaments, make them valuable models for studying human-related conditions and treatments [45]. TSC isolation was performed according to a previously described methodology [21]. Tendon tissue was dissected from flexor tendons of 6-month-old pigs, sourced from a local abattoir; tissue was washed three times in sterile PBS (Lonza) with 10% penicillin–streptomycin–amphotericin B, 1% ciprofloxacin (Sigma-Aldrich) and 1% gentamicin (50 mg/mL gentamicin, Lonza), followed by a wash in Hanks' Balanced Salt Solution (HBSS) (Lonza) with 2% PSA, 0.2% ciprofloxacin and 0.2% gentamicin. After washing, the tissue was cut into small pieces and digested with type II collagenase (Gibco) overnight at 37°C. The enzymatic activity was neutralized with foetal bovine serum (FBS) (Biosera) and tissue pieces passed through a 70-μm cell strainer (Falcon), to yield single-cell suspensions, and centrifuged at 350 RCF for 8 min at room temperature. The released cells were resuspended in DMEM—low glucose with L-glutamine and sodium pyruvate (Corning), 15% FBS, 1% PSA, 0.4 ng/mL epidermal GF (EGF) (Sigma-Aldrich), 2.5 ng/mL basic fibroblast GF (b-FGF) (Sigma-Aldrich) and 2.5 ng/mL stem cell factor (SCF) (Peprotech). To prevent mycoplasma contamination, 0.1 μg/mL mycoplasma removal agent (MRA) (Bio-Rad) was added for the first 2 weeks. Isolated cells were seeded at 5200 cells/cm^2^ and cultured at 37 C in humidified incubator at either air (21%) or physiological (2%) oxygen conditions. After 48 h of initial plating, wells were washed twice to remove the remaining nonadherent material. When cells reached confluence, they were split using 0.05% (w/v) trypsin/0.02% (w/v) ethylenediaminetetraacetic acid (EDTA) (Lonza) (5 g/L trypsin and 2 g/L EDTA diluted 1:10 in calcium-and magnesium-free PBS) and cryopreserved for subsequent use. In-depth analysis of the isolated TSC populations is described elsewhere [21].

### 2.3. MSC Growth Rate

To determine MSC long-term proliferative ability, the cells were subcultured for a period of 100 days in both 21% and 2% oxygen concentration. At each passage, cells were counted using a haemacytometer. To calculate population doublings (PDs) at the first passage, it was assumed that each cell colony originated from a single adherent human mesenchymal stem cell (hMSC), as elsewhere described [46]. PDs were calculated using the following formula:

  $$\begin{array}{c} \text{PD}=\frac{\log_{10}\left(\frac{N}{N_{0}}\right)}{\log_{10}2}, \end{array}$$

where *N* was the number of harvested cells from a T25 flask at passage and *N*_0_ was the initial number of seeded cells. The resulting values were then used to calculate the cumulative PDs (CPDs) as a function of time using the following formula:

  $$\begin{array}{c} {\text{CPD}}_{n}={\text{PD}}_{0}+{\text{PD}}_{n}+{\text{PD}}_{n+1}\ldots , \end{array}$$

where *n* is the time point which is equivalent to the days in culture.

### 2.4. MSC Immunophenotyping

In accordance with the International Society for Cellular Therapy guidelines [47, 48], we analysed the MSCs' expression of cell surface markers, CD73, CD90, CD105, CD14, CD19, CD34, CD45 and HLA-DR. MSCs at P1 were enzymatically detached and counted, and 15 × 10^4^ cells for each marker were labelled according to the manufacturer's protocol (Miltenyi Biotech). Briefly, cells were spun down at 300 RCF for 3 min and incubated for 10 min at 4°C with the directly PE-conjugated antihuman antibodies or an IgG1 isotype control. After antibody incubation, samples were washed twice with PBS and resuspended in the same buffer for acquisition. Samples were measured with CytoFlex Flow Cytometer (Beckman Coulter) and analysed with CytExpert 2.2 software. A minimum of 20,000 events were recorded. Flow cytometry events were gated by forward scatter (FSC) versus side scatter (SSC) and doublet exclusion (FSC-A vs. FSC-H). Fluorescence histograms were gated by defining the negative population based on the isotype control.

### 2.5. MSC Trilineage Differentiation

Trilineage differentiation (adipocytes, osteocytes and chondrocytes) was performed as follows: cells were seeded at 3 × 10^4^ cells/cm^2^ and cultured for 21 days in the appropriately supplemented differentiation medium. Adipogenic medium consisted of DMEM supplemented with 0.1 M dexamethasone (Sigma-Aldrich), 0.5 mM 3-isobutyl-1-methylxanthine (Sigma-Aldrich), 10 mg/mL human insulin solution (Sigma-Aldrich), 100 μM indomethacin (Sigma-Aldrich), 10% v/v FBS, 1% v/v NEAA, 1% v/v L-glutamine and 1% v/v PSA. Osteogenic medium consisted of DMEM supplemented with 50 μM ascorbic acid (Sigma-Aldrich), 10 μM β-glycerophosphate (Sigma-Aldrich), 0.1 μM dexamethasone, 10% v/v FBS, 1% v/v NEAA, 1% v/v L-glut and 1% v/v PSA. Chondrogenic media consisted of DMEM supplemented with 1% v/v insulin–transferrin–selenium (Gibco,), 0.1 μM dexamethasone, 50 μM ascorbic acid, 40 μg/mL L-proline, 1% v/v sodium pyruvate (Gibco), 10 ng/mL recombinant human TGF-β3 (Peprotech), 1% v/v FBS, 1% v/v NEAA, 1% v/v L-glut and 1% v/v PSA.

Cells were seeded on day -1 in standard culture medium. After 24 h, at day 0, standard medium was replaced with differentiation media. The media were changed twice weekly; cells at days 0, 7 and 14 were fixed with 10% formalin and stored at 4°C overlaid with PBS.

To detect osteogenesis, fixed cells were immersed in a paper-filtered solution of 2% (w/v) Alizarin Red S (Alfa Aesar) in distilled water for 10 min and washed gently with tap water. Wells were then allowed to dry before image acquisition. To detect chondrogenesis, fixed cells were stained overnight in a paper-filtered 1% (w/v) Alcian Blue (Sigma-Aldrich) in 0.1 M aqueous HCl solution. The wells were then washed gently under running tap water until clear. Stained monolayers were imaged as soon as possible thereafter. Adipogenesis was detected by first rinsing fixed cells in 60% isopropyl alcohol before staining with Oil Red O (Sigma-Aldrich) solution. An Oil Red O stock solution was prepared as a saturated solution using 300 mg of Oil Red O in 100 mL of 99% (v/v) isopropanol; to ensure maximum saturation of the solution, it was left at least overnight before being used. The working solution was prepared fresh immediately prior to staining; the stock solution was diluted to 60% (v/v) in dH_2_O and the resultant solution filtered using a 0.22-µm syringe filter. PBS covering the wells was removed, and the cell monolayer was washed rapidly with a small amount of 60% isopropanol and covered with the filtered Oil Red O working solution for 5 min, after which the stain was removed and the wells washed once with 60% isopropanol before imaging. Pictures were taken with EVOS XL Core Configured Cell Imager (Invitrogen).

### 2.6. MSC and TSC GF-Mediated Differentiation

For exogenous GDF-5-/GDF-6-/GDF-7 induced differentiation P1 cells were thawed and seeded in DMEM (4.5 g/L glucose) supplemented with 10% FBS (Lonza), 1% NEAA 1% L-glutamine (all Lonza, UK) and 50 μM ascorbic acid in 6-well plates for subsequent RNA extraction and in 48-well plates for immunofluorescence (IF) analysis. hMSCs were seeded at a density of 2.5 × 10^3^ cells/cm^2^, and TSCs were seeded at 350 cells/cm^2^. At the point of seeding, all test wells were also supplemented with 10 ng/mL of either GDF-5, GDF-6 or GDF-7 (all Peprotech). Non-GDF-supplemented cell cultures were used as a control.

### 2.7. RNA Isolation and One-Step Quantitative Reverse Transcription PCR (RT-qPCR)

Total RNA was extracted from three experimental replicates using the RNeasy Mini Kit [49] (Qiagen) according to the manufacturer's protocol. RNA concentration and quality were determined using a Nanodrop 2000 (Thermo Scientifc). Aliquots of the total RNA were stored at −80°C until further use. For RT-qPCR, only RNA samples that were determined to have a minimum optical density (OD) 260/280 of >1.8 and 10 ng of RNA were used per reaction.

RT-qPCR to determine relative expression (2^−ΔΔcT^) was undertaken using QuantiNova SYBR Green RT-PCR Kit (Qiagen), according to the manufacturer's instructions using an AriaMx Real-Time PCR Thermal Cycler (Agilent technologies). The following cycling conditions were used: reverse transcription at 50°C for 10 min, hot start at 95°C for 2 min, followed by 40 repeated cycles of amplification at 95°C for 5 s for denaturation and combined annealing/extension at 60°C for 10 s.

MSC samples were analysed using the following primers (Table 1), designed using Primer-BLAST: Scx, Tnmd, Thromb-4, Tnc-C, YWHAZ and GAPDH, all synthesized by Sigma-Aldrich. Quantitative real-time PCR was used to measure the RNA transcription level of two candidate housekeeping genes: GAPDH and YWHAZ. YWHAZ was selected as housekeeping gene, after analysing stability across the samples, and results were normalized with respect to the average expression of the housekeeping gene at the three different time points. Data are expressed with respect to the control group at day 1. To confirm the specificity of amplification products, the RT-qPCR amplicons of all the genes were separated by agarose gel electrophoresis.

To quantitatively estimate the relation between every sample in the system and study how close each data point is related to each other in the system, we applied the method of hierarchical clustering (OriginLab2023). Due to the high number of different variables analysed, the data were normalized with respect to raw, to be able to compare the single gene expression across the different samples. The distance between clustered points was calculated using the Euclidean distance. So, given the two points *P* (*x*_1_, *y*_1_) and *Q* (*x*_2_,*y*_2_), the distance is defined as follows:

  $$\begin{array}{c} d=\sqrt{{\left(x_{2}-x_{1}\right)}^{2}+{\left(y_{2}-y_{1}\right)}^{2}}. \end{array}$$

TSC samples were similarly tested with the following primers (Table 2), designed using Primer-BLAST for Sus (pig) species: Scx, Tnmd, Thromb-4, Tnc-C and Actinβ (Act-β), all synthesized by Sigma-Aldrich. Data were normalized to Actinβ.

### 2.8. IF Assay

IF was performed to evaluate Tnmd expression in MSCs and TSCs. Cells at each time point (days 1, 7 and 14) were fixed with 10% formalin; wells were then filled with PBS and stored at 4°C until staining. To stain, PBS was removed and treated with 100 µL of permeabilization buffer (0.15% Triton X-100) (Sigma-Aldrich) in PBS for 10 min. After incubation, wells were washed once with PBS and 100 µL of blocking buffer (1% BSA[10% PBS, Sigma Life Sciences + 0.1% Tween-20 in PBS) added for 1 h. After 1 h of incubation, wells were washed with PBS, treated with 100 µL of primary antibody (anti-Tnmd antibody, 1:150 dilution) (Abcam) overnight at 4°C. The following day, all wells were washed thoroughly X3 with PBS. After washing, 100 µL of the secondary antibody (Goat Anti-Rabbit IgG H&L Alexa Fluor 488 preadsorbed) (Abcam), dilution 1:500, was added to each well for 1 h at RT in the dark. After incubation, wells were washed with PBS, and 100 µL of DAPI was added for 10 min. A final wash with PBS was performed before imaging. Samples were observed with a Nikon Eclipse Ti-S fluorescence microscope, and images were captured using micro-Manager microscopy software. Semiquantitative analysis was performed with ImageJ measuring the fluorescence intensity of individual cells shown in at least three different regions of interests for any replicate.

### 2.9. Statistical Analysis

Resulted obtained from the characterization of MSCs were obtained from multiple *n* = 3 biological replicates and are represented as mean ± standard deviation (SD). A two-way ANOVA was conducted for multiple comparisons involving more than two groups of data. For comparisons between two groups, an unpaired two-tailed *t*-test was applied. Statistical analysis was performed with GraphPad Prism (version 10.3.1 for macOS, GraphPad Software, Boston, Massachusetts, USA, www.graphpad.com). Statistical significance is indicated by *⁣*^*∗*^ for *p* < 0.05, *⁣*^*∗*^*⁣*^*∗*^ for *p* < 0.01, *⁣*^*∗*^*⁣*^*∗*^*⁣*^*∗*^ for *p* < 0.001 and *⁣*^*∗*^*⁣*^*∗*^*⁣*^*∗*^*⁣*^*∗*^ for *p* < 0.0001.

## 3. Results

### 3.1. Oxygen Levels Affect MSC Morphology

**Figure 1:**
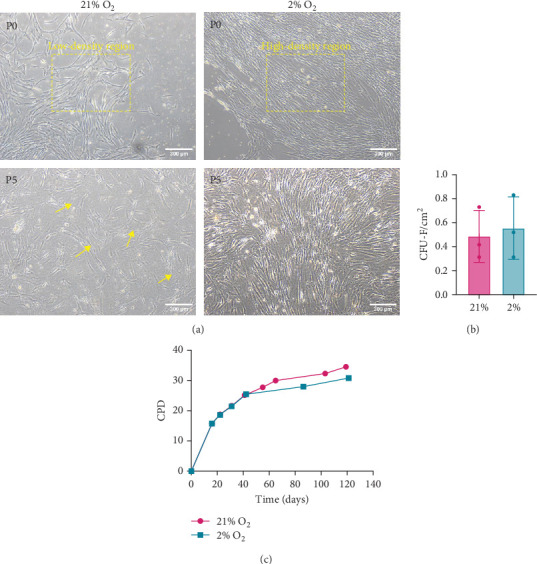
Growth potential of BM-hMSCs in different oxygen concentrations. (a) Representative image of BM-hMSCs cultured in different oxygen conditions at passage P0 and P5. (b) Clusters of cells stained positively with Giemsa, containing more than 16 cells, were considered as colony- Fforming units-fibroblastic (CFU-F). (c) Cumulative population doubling (CPD) of the BM-hMSCs. Data are presented as mean ± SD (*n* = 3). An unpaired two-tailed *t*-test was performed for statistical analysis. Differences were not significant between groups.

MSCs were isolated via adhesion to cell culture plastic and cultured in either physiological oxygen (2%) or air oxygen (21%). At P0, cells cultured in 2% O_2_ were small and demonstrated tight growth patterns establishing dense cell fields interspersed with empty areas, compared to the cells cultured at 21%, in which the cells were more uniformly distributed and less compacted. At P5, cells cultured in air oxygen consisted of heterogeneous cell populations in which cells started to acquire a large and flat morphology (yellow arrows). The cellular morphology of MSCs in 2% O_2_ was biased towards a spindle shape and less convex than air-cultured counterparts (Figure 1a).

CFU-F numbers were higher in 2% O_2_ but not at a level that reached significance (Figure 1b). MSC flasks reached confluency at P0 after 16 days in both 2% O_2_ and air oxygen. Cell growth kinetics revealed that the proliferation rate of cells obtained from both the oxygen conditions was very similar for the first 40 days, and then the rate increased gradually in air oxygen (Figure 1c).

### 3.2. MSCs Cultured Under 2% O_2_ Retain Multipotency: Immunophenotype and Trilineage Differentiation Capacity

**Figure 2:**
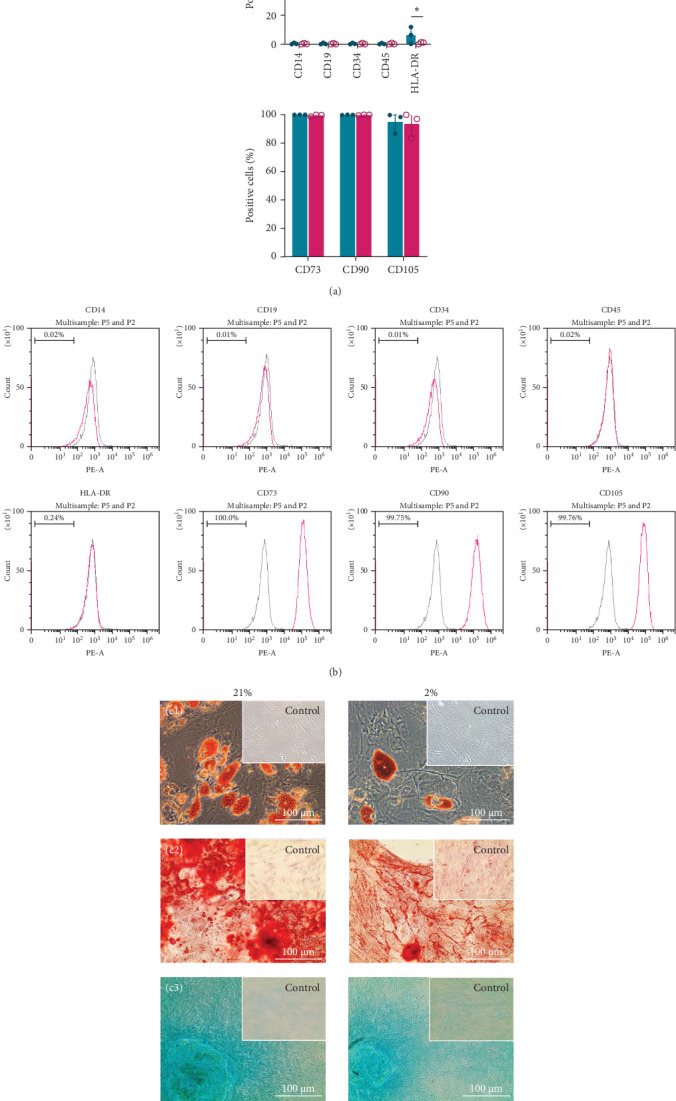
Immunophenotyping and trilineage differentiation of hMSCs. (a) Flow cytometry analysis determining the percentage of positive cell population for stem cell–specific markers. Data are presented as mean ± SD (*n* = 3). Two-way ANOVA test followed by Turkey's multiple comparison test was performed for statistical analysis. ^∗^*p* ≤ 0.05. (b) Flow cytometer histogram analysis performed with CytExpert on hPL-cultivated cells at 2% oxygen concentration. (c) hPL-cultured cells were harvested and subjected to trilineage differentiation using standard differentiation-inducing culture conditions. Adipogenic (c1), osteogenic (c2) and chondrogenic (c3) differentiation was carried out in both 21% and 2% O_2_.

Functional characterization of MSCs requires evidence of immunophenotype and differentiation capacity. Human MSCs, isolated from three independent patients, fulfilled the MSC criteria [48]: (i) typical plastic adherent growth; (ii) expression of CD73, CD90 and CD105 and absence of surface antigens CD14, CD19, CD34, CD45 and HLA-DR (Figure 2a,b); and (iii) in vitro differentiation potential towards adipogenic, osteogenic and chondrogenic lineages (Figure 2c). The primary distinction among the analysed negative markers was observed for HLA-DR expression. Specifically, cells cultured under 21% O_2_ exhibited an HLA-DR expression level that was four times higher compared to cells cultured at 2% O_2_, with a mean value of 6 ± 9%. The substantial SD observed in the 21% O_2_ group highlighted significant patient-specific variability in HLA-DR expression under air oxygen conditions, a variability not detected in cells cultured under physoxia.

Differentiation into adipogenic, osteogenic and chondrogenic cell lineages was evaluated via histological staining. Qualitative assessment of Oil Red O staining revealed that, after 21 days, adipogenic-induced MSC populations produced higher amounts of lipid droplets in 21% O_2_ (Figure 2c). Osteogenic differentiation was evaluated based on extracellular matrix calcification. Mineral calcium deposits were visualized by Alizarin Red staining and observed in all investigated groups. Noticeably greater nodules were observed in cells at 21% O_2_. Histological staining of chondrogenic samples with Alcian Blue, after 21 days of induction, showed that the samples from both oxygen concentration groups formed condensations with increased sulphated glycosaminoglycan staining. No obvious differences were observed between the groups.

### 3.3. GDF-7 Enhances the Tenogenic Differentiation of MSCs

**Figure 3:**
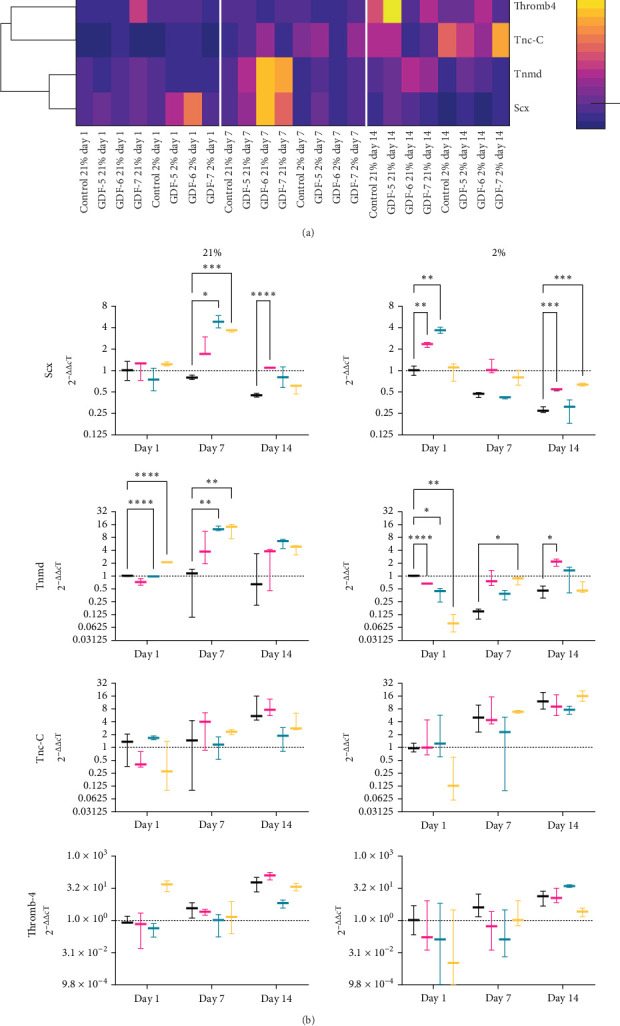
GDF-7 induces tenogenic differentiation of hMSCs. (a) Hierarchical clustering highlights the relative commitment of the different growth factors in the expression of the tenogenic genes. Dark colours indicate downregulated genes and light colours upregulated genes. (b) Box and whisker plots indicate relative gene expression. Relative gene expression was normalized to mean housekeeping gene expression. The data were expressed relatively to the untreated control sample on day 1. *n* = 3; all data represent mean ± SD. Statistical analysis has been performed with two-way ANOVA test followed by Dunnett's multiple comparison test with *⁣*^*∗*^*p* < 0.05, *⁣*^*∗∗*^*p* < 0.01, *⁣*^*∗∗∗*^*p*  < 0.001 and *⁣*^*∗∗∗∗*^*p*  < 0.0001.

We next compared the effect of supplementation with GDF-5, GDF-6 and GDF-7 in driving MSC tenogenic differentiation in both air and physiological oxygen conditions. RNA extraction was performed at three different time points, day 1, day 7 and day 14. RT-qPCR was used to analyse the expression of Scx, Tnmd, Thromb-4 and Tnc-C across all the conditions. A clustering heatmap (Figure 3a) was generated. The expression of any gene was standardized by ranking across all the samples, to compare the expression levels of each. We evinced a high correlation between the expression of Scx and Tnmd, which tended to be upregulated relatively early on day 7. This was particularly evident for cells treated with GFs and cultured in 21% oxygen. Tnc-C and Thromb-4 also showed a tendency for correlated expression, at the later day 14 time point.

Specific gene analysis is showed in Figure 3b. In both oxygen condition, Scx expression in untreated MSCs decreased over the time. Providing a GF-enriched environment was essential to stimulate MSC's expression of Scx in 21% O_2_, maintaining its expression above control samples levels. By day 7, all GF-treated samples reached a peak of maximal expression, with statistically significant results for GDF-6 (*p* < 0.05) and GDF-7 (*p* < 0.001), with sixfold and fivefold increases, respectively. At day 14, all samples showed a decrease in Scx expression compared to day 7 levels. Despite this, a significantly upregulated expression persisted in GDF-5 treated cells compared to the control group (*p* < 0.0001). Cells in 2% O_2_ had an immediate, significant upregulation in Scx expression at day 1 in response to both GDF-5 and GDF-6 (two- and fourfold increases, respectively). Thereafter, there was a decline in gene expression over time in all samples. As with 21% O_2_, GF supplementation decreased the decline in Scx expression compared to controls with the greatest effect seen at day 14 with GDF-5 and GDF-7 (*p* < 0.001).

In 21% O_2_ cultures, Tnmd expression in the untreated control group remained at baseline levels overall. When exposed to GFs, Tnmd expression was upregulated, by an increase at day 7 and a decrease on day 14. At day 1, GDF-7 treated samples showed significant upregulation (twofold increase). By day 7, there was a further increase in Tnmd expression in all treated samples, significantly so in GDF-6 and GDF-7 (*p* < 0.01), with the highest upregulation still evident in these samples at day 14. In 2% O_2_, Tnmd at day 1 was reduced in all samples exposed to GFs. Subsequently, GDF-5 and GDF-6 samples showed a gradual rise over time, achieving presupplementation levels on day 14, with a twofold and onefold increase, respectively, compared to the control group on day 1. On the other hand, GDF-7 samples showed a rise on day 7, followed by a stabilization at the same levels as the control group on day 14.

In both oxygen conditions, Tnc-C and Thromb-4 displayed a progressive increase in expression within the untreated control group over time. In 21% O_2_, on day 1, Tnc-C was increased in cells treated with GDF-6, to be then downregulated at later time points. At later time points, the expression of Tnc-C increased for GDF-5 and GDF-7 treated samples, without any significant difference with respect to the control group at that specific time point. In 2% O_2_, Tnc-C levels rise in all conditions as time passes, except for the GDF-6 sample where the high variability among experimental replicates prevents a reliable assessment of its pattern. GDF-5 and GDF-7 samples showed elevated gene expression levels over time; however, only the GDF-7 samples were found to be highly expressed compared to the control group at the same time point, despite no statistically significant differences being observed.

With Thromb-4 in 21% O_2_, both the control untreated group and the GDF-5 samples showed a gradual increase in gene expression over time, peaking at day 14 when the GDF-5 samples displayed a twofold higher expression of Thromb-4 compared to the untreated sample at that time point. GDF-6-treated samples increased Thromb-4 expression over time at a level consistently lower than the corresponding control group. GDF-7 samples displayed a distinct pattern compared to the other conditions, with the highest overexpression observed at day 1 (44-fold increase), followed by a decrease at day 7, and then increasing again at day 14. Finally, Thromb-4 in 2% O_2_ showed an increased gene expression in the control group over time. GDF-5 and GDF-6 samples were downregulated at day 1 and day 7 and reached higher values at day 14, with a onefold and threefold increase, respectively. Conversely, GDF-7 upregulated Thromb-4 at day 7, with a small increment at day 14. Despite the earlier upregulation, the samples treated with GDF-7 exhibited a lower gene expression compared to the control group at the corresponding time points. To confirm the specificity of amplification products, the RT-qPCR amplicons of all the genes were separated by agarose gel electrophoresis (Supporting Information Figure S1).

**Figure 4:**
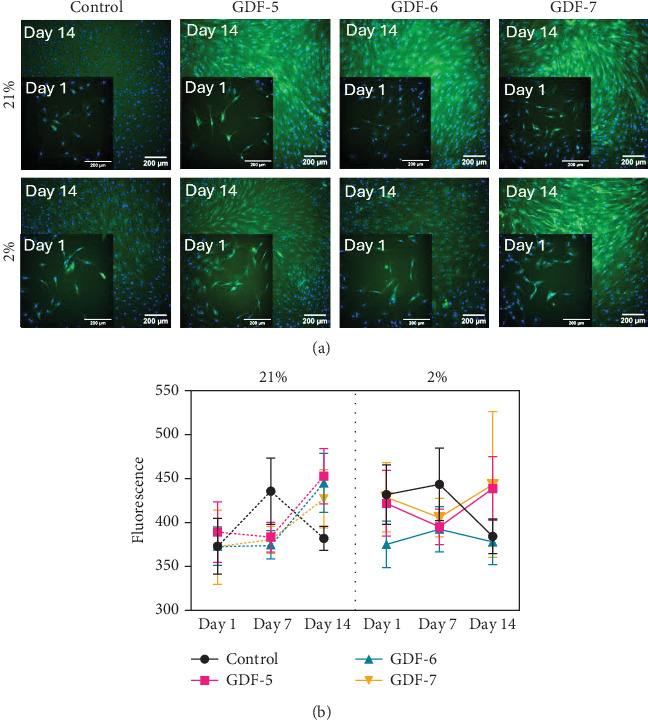
Tenomodulin expression in MSCs. (a) shows immunofluorescence images with DAPI staining in blue and Tenomodulin staining in green. Accordingly with the RT-qPCR results, in 21% O_2_, the fluorescence in GDF-5-, GDF-6- and GDF-7-treated cells is comparable. Conversely, in 2% O_2_, GDF-5 and GDF-7 seemed to improve the Tnmd expression. Grey values were used for fluorescence quantitative analysis of immunofluorescence images (b). Histograms values were fixed for all the images at 290–596 and data represented as mean ± SD.

Tnmd protein expression was evaluated in all GDF supplemented samples via IF (Figure 4). Control samples showed a consistent decrease in fluorescence by day 14, regardless of the oxygen conditions. In air oxygen, all GF-treated samples displayed fluorescence increase by day 14, with no significant variances between the GFs. In contrast, in an environment with 2% oxygen, a decrease at day 7 and then a consecutive increase by day 14 were observed with GDF-5 and GDF-7. Conversely, GDF-6 samples did not show any Tnmd increase in time.

### 3.4. Physoxia Conditions Guide TSC Differentiation Into Tenocyte

**Figure 5:**
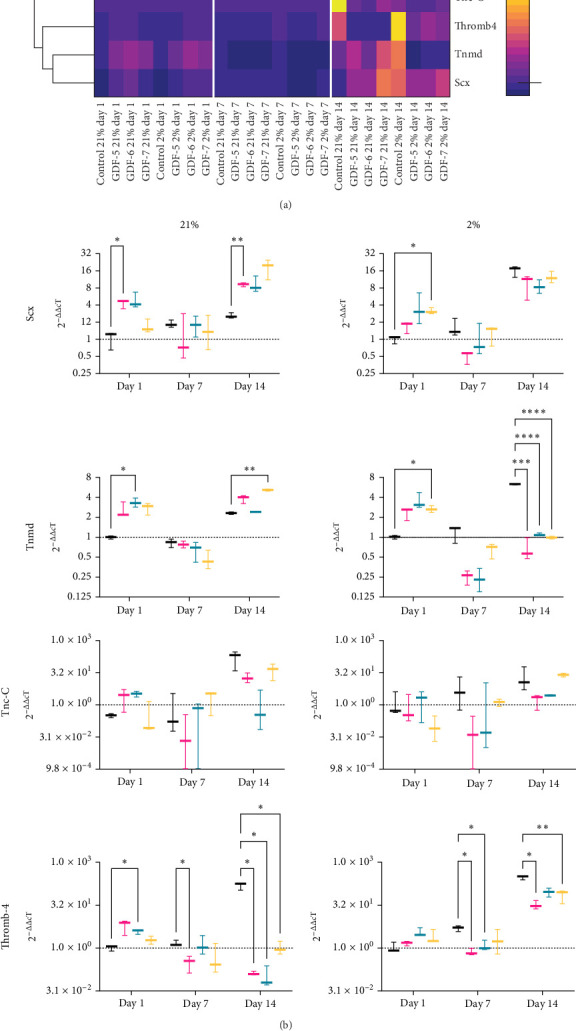
Physoxia enhances TSC tenogenic differentiation. (a) Hierarchical clustering highlights the relative commitment of the different growth factors in the expression of the tenogenic genes. Dark colours indicate downregulated genes and light colours upregulated genes. (b) Box and whisker plots indicate relative gene expression. Relative gene expression was normalized to mean housekeeping gene expression. The data were expressed relatively to the untreated control sample on day 1. *n* = 3; all data represent mean ± SD. Statistical analysis has been performed with two-way ANOVA test followed by Dunnett's multiple comparison test with *⁣*^*∗*^*p*  < 0.05, *⁣*^*∗∗*^*p*  < 0.01, *⁣*^*∗∗∗*^*p* < 0.001 and *⁣*^*∗∗∗∗*^*p* < 0.0001.

The impact of GDF-5, GDF-6 or GDF-7 supplementation on tenogenesis was similarly assessed in porcine TSCs using RT-qPCR and IF. Clustering analysis indicated Scx and Tnmd expression was also correlated in TSCs (Figure 5a). Overall, the expression of tendon-associated genes in TSCs was relatively unchanged compared to controls at day 7, followed by an increase at day 14.

In 21% O_2_, Scx expression gradually increased over time in the control group (Figure 5b). Upregulation was increased following GF supplementation at days 1 and 14 (up to sevenfold with GDF-7), achieving statistical significance with GDF-5 (threefold increase). With 2% O_2_, on day 1, the expression of Scx was elevated in the GDF-7 exposed group (*p* < 0.05), showing a threefold rise. Thereafter, no significant differences were seen in GF treated groups. In contrast to MSCs in reduced O_2_, Scx expression increased in TSCs over time to day 14; however, no significant benefit to gene expression was found with GF supplementation relative to untreated controls.

Tnmd expression in 21% O_2_ mirrored Scx expression changes, showing a decline at the midpoint followed by a rise at day 14. At day 1, all GFs showed a trend for Tnmd upregulation, with statistical significance with GDF-6 (*p* < 0.05). By day 14, all GF treated samples showed a trend for higher expression compared to controls, with the GDF-7 treated samples showing a twofold increase (*p* < 0.01) compared to the untreated control group at the same time point. In 2% O_2_ Tnmd was again upregulated in all the GFs groups, with statistically significant values for GDF-7 treated samples (threefold increase) at day 1. Subsequently Tnmd expression increased more in control samples compared to 21% cultures with GF supplementation having a negative effect on Tnmd expression levels, being significantly lower than controls by day 14 (*p* < 0.001).

Tnc-C expression in both 2% and 21% increased over time; no significant differences were seen between control and treated groups in either oxygen. In both oxygen conditions, Thromb4 demonstrated upregulation in the control group for an extended duration, with minimal correlation to the GF employed. Upregulation in the control group was significantly higher with respect to the GDF treated samples. Moreover, Tnc-C and Thromb-4 expression increased at a faster rate in the untreated control group compared to all other conditions when exposed to 2% O_2_. To investigate the specificity of amplification products, the RT-qPCR amplicon of all the genes was separated by gel electrophoresis (Supporting Information Figure S2).

**Figure 6:**
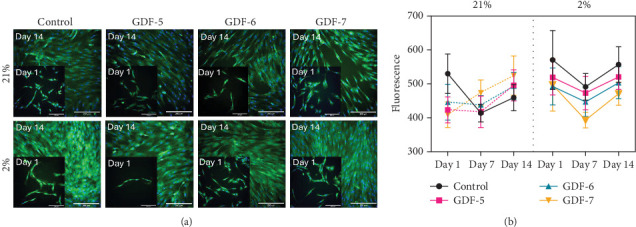
Tenomodulin immunofluorescence analysis on TSCs. (a) shows immunofluorescence images with DAPI staining in blue and Tenomodulin staining in green. At 21% oxygen concentration, the GF-supplemented media maintained expression of Tnmd; conversely, at 2% oxygen concentration, the control untreated sample is characterized by a higher MFI compared to all the other treated samples. Grey values were used for fluorescence quantitative analysis of immunofluorescence images (b). Histograms value were fixed for all the picture at 290–596 and data represented as mean ± SD.

Tnmd protein expression was further investigated by means of IF analysis (Figure 6). The results are in line with the genetic study, showing that in 21% oxygen, Tnmd fluorescence in the control group decreased on day 7 and then slightly rose on day 14. GDF-5 and GDF-6 both adhere to the same pattern. On the other hand, in the GDF-7 group, the fluorescence intensity grew throughout the entire time frame. So, the introduction of any GF, especially GDF-7 treatment, played a crucial role in maintaining a consistent expression of Tnmd under air oxygen levels. Conversely, under physoxic conditions (2% O_2_), the control group demonstrated the ability to achieve elevated Tnmd expression independently of any additional soluble signals.

## 4. Discussion

In this work, we analysed MSC differentiation into tenocytes mediated by the combined effect of GF supplemented media and variable oxygen levels. We demonstrate that this differentiation occurs as default in TSCs when they are cultured in physiological oxygen conditions. Due to a restricted amount of cells and inadequate blood supply, tendons are more prone to undergoing changes in the tissue environment following an injury, which can lead to ectopic bone formation instead of full tissue recovery. Adult stem cell application in tissue engineering has the potential to repair damaged tissues into healthy and functioning ones [50].

The application of stem cells in tendon tissue engineering offers several advantages over terminally differentiated somatic cells because of their properties and capacity to provide the necessary signals for tendon regeneration, unlike terminally differentiated cells [18]. Despite bone marrow tissue being the main source of MSCs, there is ongoing controversy regarding their usage, such as the possibility of ectopic bone formation following BMSC treatment [51]. Pretreating MSCs with specific GFs to tailor differentiation via activation of expression of specific genes associated with tenocytes could be one way to bypass this issue. TSCs have been proposed as an alternative to MSCs [52]. Until recently, tenocytes were considered to be the only cell type in tendons, while recent descriptions suggest that ~5% of tendon cells are TSCs in the tendons of humans, mice, rats, rabbits and pig [21, 22, 53]. TSCs play a crucial role in maintaining the equilibrium within tendons by possessing the ability to differentiate into specialized tenocytes in order to maintain the tissue's homeostasis. Beyond their role in maintaining tendon integrity, TSCs exhibit characteristics typical of adult stem cells, affording them a diverse range of differentiation potentials.

Multiple signalling pathways and transcription factors have been identified [54, 55] and associated with the control of tendon formation during different phases. While the roles and connections of these transcription factors remain uncertain and cannot yet be linked to form a complete pathway, there is clarity in the relationships between some factors both upstream and downstream. Through these studies, specific GFs and transcription factors that play a role in tenogenesis during development and repair processes have been identified. We examined how the concentration of oxygen and the addition of GDF-5, GDF-6 and GDF-7 to the media affected the differentiation of MSCs and TSCs towards tenocytes. We applied a concentration of GFs equal to 10 ng/mL; the dosage explored is generally recognized to have a biological action in vitro within tenogenic commitment studies, and it has been kept constant across all the GFs analysed in order to apply a more reliable comparison; higher concentrations up to 1000 ng/mL did not show improved effects and instead induced cell apoptosis and were not discussed in the this study [56].

Although many studies have used GF supplements to induce tenogenesis with varying degrees of success, their efficacy is difficult to evaluate because of the different cell types, varying durations and different readout parameters. Hence, it is necessary to compare the bioactive molecules directly in order to evaluate and determine which is the most potent in inducing tenogenic differentiation; for this reason, we decided to compare in parallel the aforementioned GFs at both the transcript and protein level. More specifically, we chose the most common tendon-related genes, namely, Scx, Tnmd, Tnc-C and Thromb-4. The results were then compared with the expression of Tnmd by means of IF imaging analysis.

In 2% O_2_, MSCs showed elevated clonogenicity, highlighted by higher CFU-F number, and concurrently, they maintained their stemness properties, as evidenced from the more gradual differentiation towards adipocyte and chondrocyte phenotypes, as opposed to the conventional 21% O_2_ environment. It has been hypothesized that culturing MSCs in physoxic conditions can result in an enhancement of multipotency and may represent a viable approach to enhance/prime the differentiation capabilities before switching to air conditions [57–59]. This idea can be associated to the stem cell niche concept, introduced for the first time by Schofield [60], that defined it as a vital microenvironment supporting stem cells in specific body locations. More specifically, stem cells cultured in oxygen levels different from those of the surrounding environment can result in changes including oxidative stress tolerance, altered metabolism, decreased growth and self-renewal, decreased ability to migrate, changes in potential to differentiate and a loss of stemness potential [61]. During tenogenic differentiation, we observed a higher expression of tenogenic genes in the 21% O_2_ group, meaning that in physiological oxygen concentration (2% O_2_), it was possible to improve the isolation but that this was refractory to the differentiation process. The transcriptional factor HIF-1α regulates crucial cellular processes such as stemness, proliferation and differentiation [62, 63]. However, while some studies suggest that under hypoxia, HIF-1α enhanced the stemness properties of MSCs while repressing their differentiation activities [59, 64], others suggest an increased differentiation potential in a HIF-1α-dependent manner [59, 65, 66]. We deduced that MSCs cultured in 2% O_2_ exhibited a slower differentiation as a consequence of a more stable stem state induced by oxygen levels; however, the precise signalling mechanisms remain to be determined.

MSCs displayed enhanced responsiveness towards tenogenic differentiation cues when in 21% oxygen culture. In the absence of GF supplementation, Scx transcript levels decreased over time, while increased expression was observed in samples treated with any of the three GFs. This confirms the requirement for biochemical stimuli to induce MSC differentiation. GDF-5, GDF-6 and GDF-7 act similarly on the transcriptional control of Scx in MSCs. Briefly, BMP ligands bind to the plasma membrane spanning type II BMP serine/threonine kinase receptors (BMPR II) which in turn binds to intracellular type I receptor (ALK2) forming an active receptor complex. This allows phosphorylation of the immediate downstream substrate proteins known as the R-Smads. Those involved in BMP signalling being Smad1, Smad5 and Smad8 (Smad1/5/8). These R-Smads then associate with Smad4, and this complex translocates to the nucleus where it functions as a transcription factor with coactivators and coexpressors to regulate gene expression [67].

Scx, expressed in the GF-treated groups, peaked at day 7 before declining at day 14, a pattern consistent with previous descriptions [68]. GDF-5, alone, upregulated Scx expression at each time point, maintaining stable expression over time. The addition of GF to the media also promoted an increase in Tnmd expression, which followed a similar pattern to Scx, reaching its highest level on day 7. As elsewhere described [19], Scx is required and sufficient for Tnmd expression. While it has been confirmed that deleting Scx results in the loss of Tnmd expression, it has also been proven that the opposite is not necessarily the case [19]. This suggests that Scx has the ability to directly promote the transcription of Tnmd. As showed by our findings, Scx and Tnmd followed similar trends across all conditions at any time point. The tight clustering of these two genes confirmed the closely connected correlation between them. GDF-7 was the most effective growth in inducing Tnmd expression, at all time points, displaying comparable efficacy to GDF-6 only on day 14. GDF-6 and GDF-7 have both been described as inducing MSC tenogenic differentiation [69, 70]. Alternate reports have detailed that silencing endogenous GDF-6 does not impact tenogenesis [71]. Conversely, silencing of GDF-7 resulted in the downregulation of expression levels of Tnmd, collagen type I and Scx. GDF-7 may therefore by a key inducer of tenogenesis including driving the expression of Tnmd in MSCs. GDF-7 has been used as a supplement in both in vitro and in vivo conditions, and it has been shown that it is a highly effective inducer of tenocyte-like cell differentiation in MSCs that appears to be sustained without the need for further exposure to exogenous GFs [19]. We are unaware of evidence to suggest that GDF-7 supplementation induces its own synthesis in MSCs. We hypothesize that direct cellular responses to GDF-7 are intrinsically long-lived, possibly reflecting changes that are characteristic of a new state of differentiation [72].

In physoxia, all groups analysed consistently showed a decrease in Scx and Tnmd expression, consistent with the observation that this can be refractory to MSCs differentiation abilities. In both oxygen conditions, the levels of Tnc-C and Thromb-4 expression gradually rose over time in the unsupplemented groups. Tnc-C, an ECM glycoprotein that interacts with various surface receptors, such as integrins and other ECM components, influences cell–matrix interactions [73]. Tnc-C is present in mature tendons playing a role in aligning collagen fibers [73]. Thromb-4 is also present in the ECM, simultaneously engaging multiple cellular receptors and ECM ligands to organize ECM and its interaction with cells [74]. Although primarily found in tenocytes, Tnc-C and Thromb-4 also aid in organizing the ECM of many tissues, and for this reason, the increased levels by day 14 of these two genes in the control group of MSCs may be attributed to an internal organization of the cell–ECM structure at confluence, rather than a specific differentiation process. When exposed to air, every GF was associated with a consistent gene expression pattern for both, indicating a correlation, although not necessarily a direct cause-and-effect relationship.

Regarding the quantitative IF assay, the commitment of MSCs towards tenocytes was investigated by means of relative Tnmd fluorescence intensity. In an oxygen environment of 21%, all three GFs triggered the activation of Tnmd. However, in 2% oxygen, where differentiation was not as effective at the genetic level, only samples treated with GDF-7 exhibited increased fluorescence values at the endpoint, underscoring the strong tenogenic differentiation capabilities of this particular GF.

To the best of our knowledge, in the context of TSCs, the impact of GDF-6 and GDF-7 has not been previously documented, whereas there are studies involving GDF-5. Holladay et al. [75] investigated TSC treatment with GDF-5, focusing on the lost of multipotency of these cells, and the expression of tendon-related genes. Treatment with GDF-5 resulted in the loss of potential for differentiation into adipocytes and chondrocytes, along with a notable rise in Scx expression at day 16. Given that this research was carried out in the presence of atmospheric oxygen, it validates the findings from our study: on day 14, GDF-5 supplementation was associated with an increased expression of Scx compared to the untreated control group. Nevertheless, by examining various timepoints, we were able to provide a clearer picture. The outcome of this more thorough analysis revealed that samples treated with GDF-5 in air experienced some fluctuation between day 1 and day 14, with a downregulation at day 7. The same pattern was observed with the Tnmd expression, under all conditions. When only looking at the last time point in 21% O_2_, GDF-7 treatment resulted in the highest expression of both Scx and Tnmd. Regarding Tnc-C and Thromb 4, a diminished expression was noted at the end point, even when compared to the control group, reassessing the hypothesis of a natural overexpression related to the confluency of the cells.

In comparison, in 2% O_2_, the control group displayed inherent overexpression of all tenogenic genes. This was consistent with Tnmd IF, where it has been shown an increase over time in cells treated with GDF-7 in air, while the control group consistently exhibited the highest Tnmd expression in physoxia. Considering that tendon is a relatively avascular, it is possible to hypothesize that physoxia is sufficient to guide the differentiation of TSCs into tenocytes. This confirms previous reports where physoxia increased tenocyte proliferation and TSC rejuvenation [76, 77]. For example, TSCs cultivated in culture medium-conditioned, from young TSCs, underwent to a consistent reduction of senescence-associated β-galactosidase activity [76]. Additionally, there was an increase in the levels of Tnmd and Tnc-C expression, indicating that transferring aged TSCs to a youthful normoxic culture medium could preserve their ability to differentiate into tendon cells. Moreover, Lee et al. [78] found that TSCs cultured at 2% O_2_ tension exhibited decreased collagenous protein production and increased mRNA level of Tnmd compared to those cultured in air. This indicates that maintaining TSCs at 2% O_2_ tension could promote tenogenic differentiation potential and suppress nontenogenic differentiation in vitro spontaneously.

## 5. Conclusion

MSCs are widely researched in tendon regenerative medicine because they can differentiate, regulate the immune system and secrete GFs. Nevertheless, there are instances in which MSCs can result in abnormal bone growth both in vitro and in vivo. To prevent this, prior treatment with GFs may enhance tenogenic differentiation. Alternatively, TSCs can express genes related to tendons and hold the ability to differentiate into tenogenic cells without requiring pretreatment. This makes TSCs a favourable choice for tendon repair in comparison to MSCs.

This study focused on the potential contribution of MSCs and TSCs in the repair and regeneration of tendons, highlighting the influence of GFs and oxygen levels on the differentiation of both cell types into tenocytes. Among the different GFs studied, GDF-7/BMP-12 was demonstrated to be an inducer of MSCs' tenogenic differentiation in 21% O_2_, suggesting its potential application in advanced tissue engineering methods. In contrast, with TSC physoxic cell culture served as an effective environmental factor for guiding differentiation of these cells into tenocytes.

This study directly compared various GFs in controlled oxygen environments. The goal was to analyse their effectiveness in promoting tenogenic activity in both MSCs and a cell line more committed to tenogenesis, such as TSCs. The complexity of MSC and TSC differentiation pathways was emphasized by our findings and established the basis for future tissue regeneration methods utilizing both environmental and biochemical control factors.

## Figures and Tables

**Table 1:** Primer sequences and conditions for RT-PCR in MSCs.

Gene	Sequence	Tm (°C)
Scleraxis	F 5′-CAAACAGATCTGCACCTTC-3′	58.4
	R 5′-CGAATCGCTGTCTTTCTG-3′	59.7
Tenomodulin	F 5′-ACATGGAAATTGATCCTGTG-3′	59.8
	R 5′-TCTCATCTATTTCCTCTTCTGG-3′	59.0
Thrombospondin-4	F 5′-CAGGGTACGATTTTATGAAGG-3′	59.9
	R 5′-TTCTGGGTTTGAAACTCTTG-3′	59.3
Tenascin-C	F 5′-GAATCTTTGCAGAGAAAGGG-3′	60.4
	R 5′-AAGTCTCTTGGAGAATCGAG-3′	57.6
GAPDH	F 5′- CTTTTGCGTCGCCAG-3′	60.3
	R 5′- TTGATGGCAACAATATCCAC-3′	60.8
YWHAZ	F 5′- AACTTGACATTGTGGACATC-3′	57.3
	R 5′-AAAACTATTTGTGGGACAGC-3′	58.2

**Table 2:** Primer sequences and conditions for RT-PCR in TSCs.

Gene	Sequence	Tm (°C)
Scleraxis	F 5′- FCAGCAGCACCTGTAACCCAAG -3′	57.4
	R 5′- AGACTCGTGGGGACGAAGA -3′	58.6
Tenomodulin	F 5′- AGAAGACCCGTCATGCCAGA -3′	60.8
	R 5′- AAGGAGCAGTGAGTTTTGCGA -3′	60.0
Thrombospondin-4	F 5′- ACCAGGGTACATCGGGATCA -3′	60.8
	R 5′- GCAAGGGTTCAGCTCTGGAT -3′	60.4
Tenascin-C	F 5′- AGATCTCGATTCTCCAAGAG -3′	60.4
	R 5′- CCGTCAACAGATTCATACAC -3′	58.6
Actinβ	F 5′- GCTAAGGGGGCGCTCTGTC -3′	60.4
	R 5′- GTGTTGGCGTAGAGGTCCTTC -3′	60.9

## Data Availability

The data that support the findings of this study are available from the corresponding author upon reasonable request.

## Supporting Information

Supporting Information 1 MSC agarose gel electrophoresis analysis. Gel electrophoresis was carried out to verify the size of the amplicon for every gene studied in MSCs following RT-qPCR.

Supporting Information 2 TSC agarose gel electrophoresis analysis. Gel electrophoresis was carried out to verify the size of the amplicon for every gene studied in TSCs following RT-qPCR.
