# Supplementary material for: Tenogenic Cues Are Biochemically and Environmentally Distinct for Tendon Stem Cells and Mesenchymal/Stromal Stem Cells
Source: Stem Cells Int. 2025 May 13;2025:9047956. doi: 10.1155/sci/9047956 (PMC12092158; doi:10.1155/sci/9047956)
Supplement: Supporting Information 2 — TSC agarose gel electrophoresis analysis. Gel electrophoresis was carried out to verify the size of the amplicon for every gene studied in TSCs following RT-qPCR. [file 9047956.f2.pptx]

## Slide 1
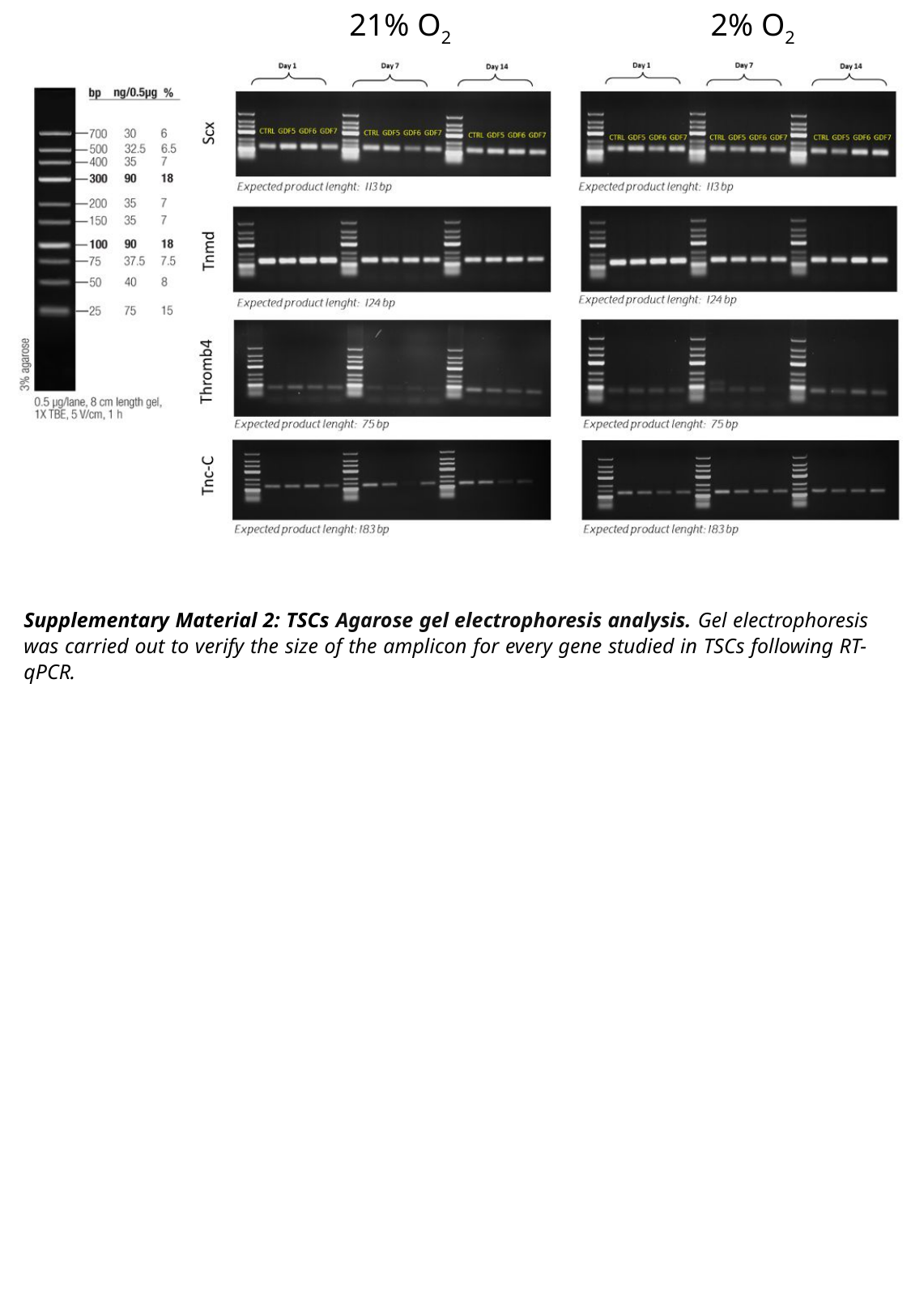

21% O2
2% O2
Supplementary Material 2: TSCs Agarose gel electrophoresis analysis. Gel electrophoresis was carried out to verify the size of the amplicon for every gene studied in TSCs following RT-qPCR.
